# Models for calcific aortic valve disease in vivo and in vitro

**DOI:** 10.1186/s13619-024-00189-8

**Published:** 2024-03-01

**Authors:** Zijin Zhu, Zhirong Liu, Donghui Zhang, Li Li, Jianqiu Pei, Lin Cai

**Affiliations:** 1 State Key Laboratory of Biocatalysis and Enzyme Engineering, School of Life Science, National & Local Joint Engineering Research Center of High-Throughput Drug Screening Technology, Hubei University, Wuhan, 430062 China; 2 State Key Laboratory of Cardiovascular Disease, Fuwai Hospital, National Center for Cardiovascular Diseases, Chinese Academy of Medical Sciences and Peking Union Medical College, Beijing, China; 3 Department of Biochemistry and Molecular Biology, School of Basic Medical Sciences, Beijing Key Laboratory of Metabolic Disorders Related Cardiovascular Disease, Capital Medical University, Beijing, 100069 China

**Keywords:** Calcific aortic valve disease, Animal model, In vitro, 3-dimentional culture

## Abstract

Calcific Aortic Valve Disease (CAVD) is prevalent among the elderly as the most common valvular heart disease. Currently, no pharmaceutical interventions can effectively reverse or prevent CAVD, making valve replacement the primary therapeutic recourse. Extensive research spanning decades has contributed to the establishment of animal and in vitro cell models, which facilitates a deeper understanding of the pathophysiological progression and underlying mechanisms of CAVD. In this review, we provide a comprehensive summary and analysis of the strengths and limitations associated with commonly employed models for the study of valve calcification. We specifically emphasize the advancements in three-dimensional culture technologies, which replicate the structural complexity of the valve. Furthermore, we delve into prospective recommendations for advancing in vivo and in vitro model studies of CAVD.

## Background

Calcific aortic valve disease (CAVD) is a chronic condition characterized by the hardening and calcification of the aortic valve leaflets (Voicu et al. 2023). Its prevalence increases with age, affecting over 25% of individuals aged 65 and older, and surpassing 50% in those over 85 (Kraler et al. 2022). At early stage CAVD, non-symptomatic thickening and sclerosis of valve leaflets do not necessitate immediate intervention. However, severe calcification, termed aortic stenosis, leads to a significant reduction in blood flow, requiring surgical intervention to replace the damaged valve with prosthetic valves, including mechanical valves, as well as biological valves derived from animal or human tissue (Lindman et al. 2013). Although surgical interventions are effective, they are associated with complications and suboptimal long-term outcomes, as there are currently no medical therapies available. Recognizing the urgency to enhance our understanding of CAVD pathogenesis and identify suitable therapies, various models have been proposed in recent years to facilitate the exploration of the underlying mechanisms of CAVD. This review aims to succinctly outline the strengths and weaknesses of these models, providing insights into their utility for CAVD research, and discussing potential future directions.

## Pathophysiology of CAVD

Calcific aortic valve disease is now recognized as an active process rather than a passive one, which is characterized by the accumulation of calcium deposits and fibrosis in the aortic valve leaflets (Wang et al. 2021a, b), leading to stiffening, narrowing, and eventually, obstruction of blood flow through the valve (Moncla et al. 2023). The pathophysiology of CAVD is complex and multifactorial, involving a combination of genetic and environmental factors, as well as various cellular and molecular mechanisms (Li et al. 2013). This understanding has been supported by research findings that demonstrate the active involvement of valvular interstitial cells in the development of CAVD. These cells undergo osteoblast-like differentiation, leading to extracellular matrix remodeling, collagen deposition, and ultimately, the formation of bone-like structures within the valve. Additionally, genetic variations in certain genes have been associated with an increased risk of CAVD, although they explain only a limited number of cases. The interplay between genetic factors, environmental factors such as hypertension and hyperlipidemia, and cellular processes contributes to the complex pathogenesis of CAVD.

### Risk factors of CAVD

Bicuspid aortic valve (BAV) is the most prevalent congenital heart defect, primarily linked to genetic factors and identified as a congenital risk factor for CAVD (Moncla et al. 2023; Yoon et al. 2020). Genomic studies have revealed associations between BAV and *NOTCH1 *(Debiec et al. 2022), *SMAD6 *(Kloth et al. 2019), *ADAMTS19 *(Ackah et al. 2023), gene members of *GATA *(Gharibeh et al. 2018) and *ROBO *(Mommersteeg et al. 2015) families. Furthermore, *NOTCH1 *(Majumdar et al. 2021)*, LPA *(Boffa & Koschinsky 2019; Smith et al. 2014)*, PALMD *(Wang et al. 2022)*, IL6* and *FADS1/2* are implicated in the initiation and progression of CAVD. However, genetic variations within these genes explain only a restricted number of cases and contribute to a moderate to low population-attributable risk. Additional research is necessary to identify novel gene candidates and expand our understanding of the genetic factors influencing CAVD.

The susceptibility of CAVD is higher in the elderly and among men. Additionally, hypertension is associated with valvular calcification regardless of age; individuals with obesity, kidney dysfunction and mineral metabolism are more likely to develop valve calcification. Abnormal blood flow disrupts tissue balance by triggering inflammatory and fibrotic signals, thereby accelerating the progression of calcific aortic valve disease and ultimately leading to the development of aortic stenosis (Kraler et al. 2022). Lipoprotein(a) fuels this process by promoting aortic valve endothelial dysfunction, inflammation, and oxidative stress in the valve (Boffa & Koschinsky 2019). Notably, oxidized phospholipids, converted by autotaxin into lysophosphatidic acid, trigger pro-inflammatory and procalcific responses through lysophosphatidic acid receptors (Pantelidis et al. 2023). Moreover, chronic renal disease and downstream vascular disorders are associated with an elevated susceptibility to CAVD (Vavilis et al. 2019). These risk factors can interact with each other, further amplifying the risk of developing CAVD.

### CAVD pathophysiology

The normal aortic valve comprises three leaflets anchored to the fibrous ring at the left ventricle's outlet. These leaflets consist of fibrous, spongiosa, and ventricularis layers, which are populated with valve interstitial cells (VICs), with the entire structure wrapped by valve endothelial cells (VECs) (Voicu et al. 2023). Lamina fibrosa facing the aorta is rich in collagen fibers, lamina spongiosa layer is composed of loose connective tissue rich in glycosaminoglycans, and lamina ventricularis oriented towards the ventricles is characterized by radially distributed elastic fibers (Jana et al. 2019). Under physiological conditions, the spongy layer of the aortic valve maintains the proper arrangement of the collagen layer (fibrous layer) and elastic layer (ventricular layer) (Tseng & Grande-Allen 2011). The gaps in the spongy layer make it highly shockproof and lubricate the two adjacent layers at the same time. Due to the presence of elastin fibers, the ventricular layer reduces radial strain, making the valve elastic (Kraler et al. 2022). The valve is extremely elastic and compressible, providing the biomechanical properties required to sustain repetitive cyclic strain over time.

The underlying pathological processes of CAVD involve dysfunction of VECs chronic inflammation, lipid deposition, remodeling of the extracellular matrix, and ectopic calcification primarily caused by VICs osteodifferentiation (Kraler et al. 2022; Moncla et al. 2023). Healthy VECs maintain an endothelial barrier ensuring optimal mating surfaces (Vesely & Noseworthy 1992). Atherogenic factors or increased mechanical stress lead to VECs injury, disrupting the endothelium, promoting oxidatively modified lipid uptake, and activating inflammation-calcification loops within VICs. VICs then differentiate into myofibroblasts and osteoblasts, triggering extracellular matrix remodeling, collagen deposition, nucleation loci formation, and ultimately, VICs-mediated bone formation (Decano et al. 2022). Understanding VICs' intricate differentiation between myofibroblast and osteoblast pathways is crucial in modeling calcific aortic valve disease.

Recent studies have shown that the pathological changes of CAVD are similar to atherosclerosis in the early stage, and may be similar to bone formation in the late stage (Yip & Simmons 2011). However, clinical patient detection and diagnosis, as well as current basic research, mostly focus on changes in the late stages of the disease, and it is still difficult to carry out the development of the early and progressive stages of the disease. This is partly due to the lack of suitable animal models of CAVD.

## Animal models of CAVD

Animal models, aside from defined traits obtained from clinical patients with CAVD, are the most commonly used method for understanding its pathological processes. Animal models like mice (Gollmann-Tepekoylu et al. 2023), rabbits (Liu et al. 2020), and porcine have been extensively discussed. Aortic valve calcification, assessed through histological techniques such as alizarin red, von Kossa or movat pentachrome staining, represents a prominent phenotype. Evaluation using imaging modalities like echocardiography and micro-CT aids in assessing valve characteristics, calcification, and hemodynamic changes (Ahmad et al. 2023). Additionally, molecular markers like Runx2 (Yu et al. 2018), osteopontin (OPN) (Passmore et al. 2015; Rajamannan et al. 2003), BMP2 (Gomez-Stallons et al. 2016), inflammatory cytokines IL-1β, IL6, and TNF-α (Combi et al. 2023) serve as crucial indicators in CAVD models. Our analysis delves into diverse methodologies utilized for modeling CAVD, while assessing the strengths and limitations inherent in each approach. The advantages and disadvantages of CAVD animal models are listed in Table 1.

**Table 1:** Advantages and disadvantages of CAVD animal models

Types of animal models	Treatment & Study (Part)		Advantages	Disadvantages
Diet-induced animal models	mouse	42% fat, 0.15 ~ 0.2% cholesterol, 1.2% choline, 2 ~ 16 m (Aikawa et al. 2009; Hakuno et al. 2010; Jung et al. 2015; Li et al. 2022; Matsumoto et al. 2010; Miller et al. 2010; Nigam & Srivastava 2009; The et al. 2022; Towler et al. 1998; Yanget al. 2021; Zeadin et al. 2009)	susceptible to genetic manipulation; Relatively cheap and easy to house; Short lifespan	Limited similarity to human disease; Small size
	rabbit	0.5% ~ 2% cholesterol, VitD2 25,000/50,000/100,000 IU /day, 2 ~ 12 w (Arishiro et al. 2007; Choi et al. 2021; Cimini et al. 2005; Drolet et al. 2008; Gkizas et al. 2010; Haberland et al. 2001; Hamilton et al. 2011; Liberman et al. 2008; Marechaux et al. 2009; Ngo et al. 2011; Rajamannan et al. 2005; Zeng et al. 2007)	Rapid disease progression; Small size and ease of handling; Availability of reagents and tools; Amenable to genetic manipulation	The anatomy and hemodynamics of rabbit hearts differ from those of humans; challenging to control experimental variables
	swine	12 ~ 15% lard, 1.5% cholesterol, 32% fat, 2 ~ 6 m (Guerraty et al. 2010; Sider et al. 2014; Simmons et al. 2005)	Similar in size, structure, function, and hemodynamics to human hearts; Spontaneously develop CAVD similar to humans	Limited availability of standardized models; Challenging to control experimental variables; Potential for species-specific differences
Genetically modified animal models	Apoe − / − mice (Aikawa et al. 2009; Hjortnaes et al. 2010; Srivastava et al. 2011; Tanaka et al. 2005; Zeadin et al. 2009); LDLr(-/-)mice (Dharmarajan et al. 2021; Gao et al. 2021; Liu et al. 2022; Schlotter et al. 2012); klotho-deficient mice (Wirrig et al. 2015)		enabling mechanistic and therapeutic target discovery; Mimics calcification & valvular dysfunction	Expensive and time-consuming; Off-target effects; Limited scope
Mechanical injury models	A direct balloon injury to the aortic valve (Kim et al. 2023); Aortic valve wire injury (AVWI) (Honda et al. 2014; Iqbal et al. 2023; Li et al. 2023; Peng et al. 2022; Toshima et al. 2020; Zhao et al. 2022; Zhong et al. 2023)		Controlled injury; Study of acute responses	Limited relevance to human pathophysiology; Lack of disease progression; Ethical considerations; Limited variability and standardization

### Diet-induced animal models

Diet-induced animal models are vital in understanding CAVD pathogenesis. High-fat or cholesterol-rich diets induce systemic inflammation and pathological changes similar to human CAVD. Rodents and large animals possess distinct characteristics when subjected to high-fat/high-cholesterol diets. Mouse aortic valve tissue lacks the requisite trilayer structure for spontaneous calcification. Despite this, mice are predominantly chosen due to their small size, ease of maintenance, and genetic manipulability in modeling CAVD (Ahmad et al. 2023). While a high-cholesterol diet can cause lipid accumulation and macrophage infiltration in mouse valves (Li et al. 2022; The et al. 2022), their relevance is constrained by prolonged duration and limited clinical significance. Therefore, the most commonly employed approach involves combining a high-fat diet with genetic or other alterations to establish a CAVD model in mice.

Large animal models like rabbits exhibit a trilayer valve shape, making them suitable for simulating CAVD symptoms through dietary cholesterol. A high-cholesterol and vitamin D supplemented diet in rabbits triggers aortic valve stenosis, calcium deposition, and elevates DPP-4 activity (Choi et al. 2021; Sider et al. 2014). However, differences in lipid metabolism between humans and rabbits, extended experimental duration, and the necessity of Vitamin D for advanced CAVD stages are limitations. On the other hand, porcine models closely resemble human heart anatomy and lipid metabolism (Schuster et al. 2010). Under high-fat/cholesterol diets, pigs develop advanced aortic stenosis (AS) stages involving necrotic core, fibrous cap, hemorrhage, calcification, and medial thinning (Wang et al. 2002). Nonetheless, the pig model's drawbacks include prolonged experimental timelines and high economic costs.

### Genetically modified animal models

Genetically modified animal models serve as crucial tools to replicate human disease traits, aiding the study of underlying mechanisms and therapeutic avenues. *ApoE* −/− and *Ldlr* −/− mice are frequently utilized for CAVD modeling due to genetic modifications (Wang et al. 2020; Weiss et al. 2006). Noteworthy genes like *Notch1*, *Postn,* associated with congenital bicuspid aortic valve, aid in studying valve development and the CAVD process using genetically modified mice (Cheng et al. 2021; Gomez-Stallons et al. 2016). Wirrig et al. demonstrated in klotho-deficient mice that the activation of BMP signaling and the induction of osteochondrogenic genes precede and localize with aortic valve calcification, highlighting the essential role of BMP signaling in the development of CAVD in vivo (Wirrig et al. 2015). Beyond mice, Watanabe heritable hyperlipidemic (WHHL) rabbits (Rajamannan et al. 2005) and pigs with mutated *LDLR* and/or apolipoprotein genes (Grunwald et al. 1999; Prescott et al. 1991) are also employed in CAVD research. These models offer valuable insights into disease mechanisms and potential therapeutic targets. In genetic editing, mice hold an edge due to their clear genetic background, facilitating simpler gene editing and benefiting from a more mature antibody system in research. Conversely, large animals have complex genomes, posing challenges for transgenic manipulations. A few strains are derived from spontaneous formation, for example, pigs with hypercholesterolemia and aortic stenosis share several traits with human CAVD (Sider et al. 2014; Skold et al. 1966). The natural Nox2 inhibitor, celastrol, was found to effectively alleviate CAVD by inhibiting Nox2-mediated glycogen synthase kinase 3 beta/β-catenin pathway in aortic valvular interstitial cells (AVICs), and was also found to reduce ROS production, fibrosis, and severity of aortic stenosis in a rabbit CAVD model (Liu et al. 2020).

### Mechanical injury models

Mechanical injury models involve applying stress or damage to valve leaflets, initiating an inflammatory response and pathological tissue remodeling leading to CAVD. A commonly used model is employing balloon catheters or wire probes to induce damage, as observed in rabbit models (Kim et al. 2023). Iqbal et al. utilized a mouse model deficient in sortilin and subjected it to aortic valve (AV) wire injury (AVWI) to investigate the impact of sortilin on AV stenosis, fibrosis, and calcification (Iqbal et al. 2023). Honda et al. (Toshima et al. 2020) induced aortic valve injury in male C57/BL6 mice by inserting and maneuvering a spring guidewire under echocardiographic guidance through the right common carotid artery into the left ventricle. Serial echocardiographic assessments revealed a significant increase in aortic velocity one-week post-injury, persistently elevated until 16 weeks’ post-injury. AVS mice showed a higher heart weight/body weight ratio, decreased cardiac function, increased valve leaflets proliferation, inflammatory cytokines and osteochondrogenic factors within 4 weeks post injury. Alizarin red staining showed valvular calcification 12 weeks after injury (Honda et al. 2014).

While each model describes different aspects of CAVD development, no single animal model consistent completely represents human CAVD with its wide spectrum of risk factors and heterogeneity. Therefore, it is recommended to use orchestrated combination of multiple animal models with in vitro studies to demystify CAVD mechanisms further and develop effective therapeutic interventions for patients with CAVD.

## In vitro models

**Fig. 1:**
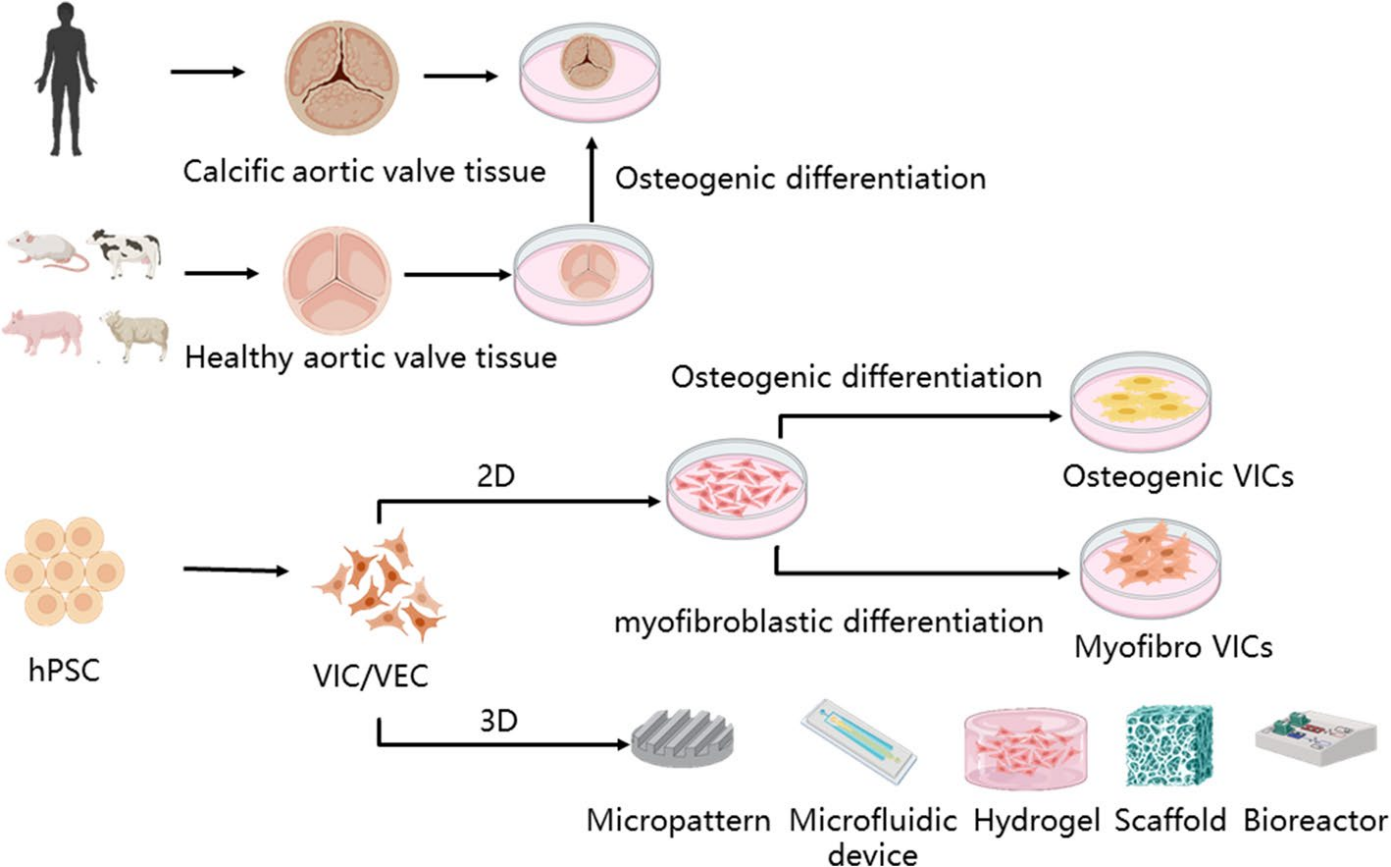
Different types of in vitro CAVD models. The most straightforward, but challenging, approach is to isolate calcified aortic valves from CAVD patients or animals. Alternatively, healthy valves can be induced using osteogenic medium. VICs and VECs are more common cell types used for in vitro CAVD modeling. These cells, isolated from healthy animals or differentiated from hiPSCs, are then treated with osteogenic or myofibrotic medium to mimic the pathophysiological processes of CAVD. In addition, 3D culture systems including micropatterns, microfluidic devices, bioreactors, hydrogels, and scaffolds can be employed to mimic the aortic valve structure, function, and complex environmental stresses associated with CAVD

With regard to in vitro models, mimicking aortic valve structure and modeling the progression of CAVD dynamics are key issues that need to be addressed. We will discuss in vitro CAVD models from plane cell model, 3 dimensional (3D) cell culture model, as summarized in Fig. 1.

### 2D cell culture models

There exist various in vitro cell models designed for investigating CAVD, categorized into two primary strategies: 1) Isolation of aortic valve cells from calcific aortic valves across different species, encompassing samples from CAVD patients. 2) Induction of calcification through osteogenic differentiation medium applied to VICs. Regarding the latter approach, several factors are usually taken into account, such as the heterogeneity of aortic valve cells, the authenticity of both external and internal stimuli, and the configuration that best mimics valve morphology or function.

#### Cell resources

##### Primary cells

The isolation of VECs and VICs is an essential step in studying CAVD in vitro. VECs are prone to contamination with VICs, necessitating further enrichment through cell sorting methods (Gould & Butcher 2010). VICs are crucial contributors to the pathogenesis of calcific aortic valve disease by transforming into activated myofibroblasts-like cells. These myofibroblast-like cells play dual roles in the disease process: they synthesize and remodel the extracellular matrix (Gee et al. 2021), while also undergoing differentiation into osteoblast-like cells, promoting calcium deposition (Hjortnaes et al. 2015). VICs can be derived from either calcified or healthy, non-calcified valves. VICs obtained from calcified valves, sourced from patients undergoing valve replacement surgery, offer a direct examination of pathological phenotypes compared to VICs from normal valves (Duan et al. 2013; Li et al. 2013). While human valves are preferred to eliminate species differences, acquiring valve cells from healthy individuals is challenging (Ferdous et al. 2011). Obtaining primary aortic valve cells from mammals, such as sheep (Immohr et al. 2022; Weber et al. 2021), porcine (Bramsen et al. 2022; Hjortnaes et al. 2016; Meerman et al. 2021), bovine (Wang et al. 2021a, b) and mice (Lim et al. 2016), are more straightforward compared to human aortic valve cells. However, these cells only partially recapitulate the human CAVD procedures. The most practical approach for in vitro studies is to isolate viable VICs from valves of healthy animals and then subject them to an activating and osteogenic differentiation medium to induce calcification.

##### hPSC-derived valvular cell model

Human-induced pluripotent stem cells (hiPSCs) have revolutionized the field of regenerative medicine by enabling the production of robust functional cells that were previously difficult to obtain, such as cardiomyocytes (Yoshida & Yamanaka 2017), neural cells (Chang et al. 2019), and hepatocytes (Ramli et al. 2020). This remarkable ability extends to the generation of VECs and VICs, offering a promising source of cells for heart valve research and therapy.

**Fig. 2:**
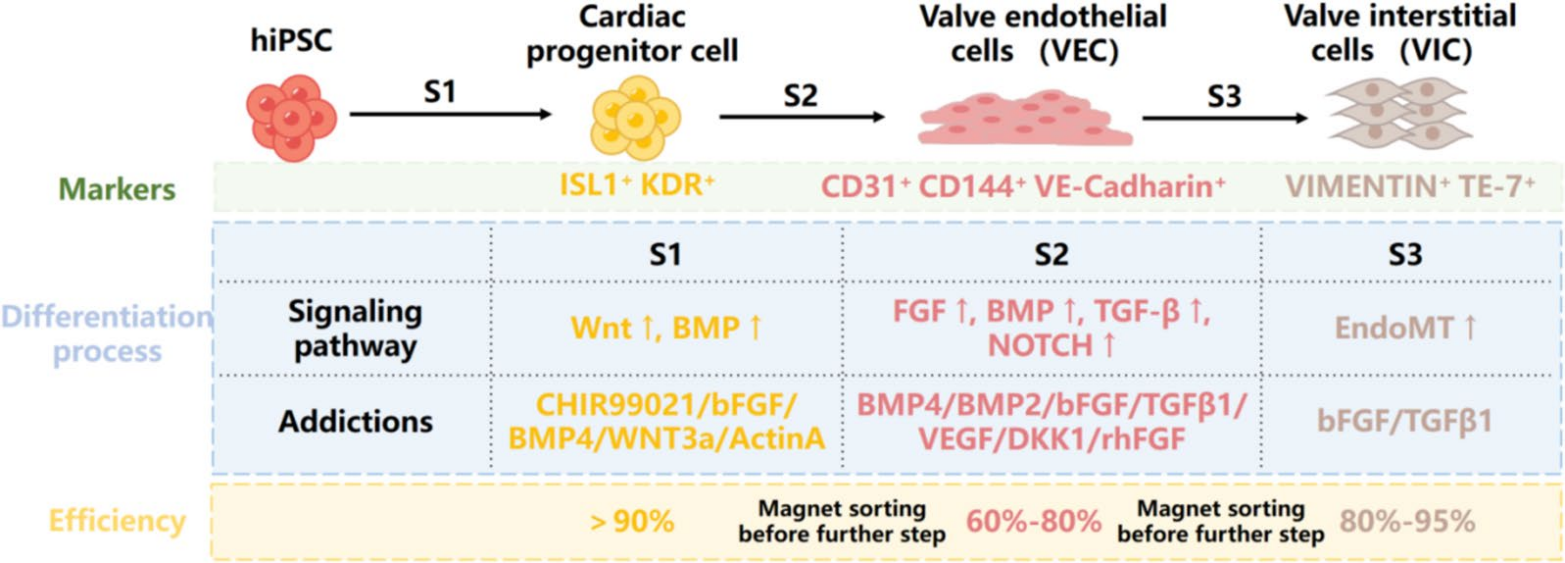
Strategy of hiPSC-derived valve endothelial cells and valve interstitial cells. hiPSC differentiation into VICs was achieved through a three-stage approach. Stage 1 involved mainly WNT signaling activation, promoting cardiac progenitor cell specification. Stage 2 employed BMP signaling activation for VEC generation. Finally, endothelial-mesenchymal transition was induced to obtain VICs. Magnetic bead sorting was implemented at each stage to enhance VIC purity and homogeneity

The current strategy for directed differentiation of hiPSCs into valve cells is a three-stage process (Cheng et al. 2021), as illustrated in Fig. 2. While different differentiation protocols employ varying inducer molecules, they all converge on the goal of recapitulating the early stages of valve formation and specialization during heart development (Armstrong & Bischoff 2004). Pluripotent stem cells (PSCs) are differentiated into mesodermal cardiac progenitor cells (CPCs) using a combination of factors, including WNT agonists (WNT3a or CHIR99021), BMP4, and bFGF. This approach has been shown to generate ISL1^+^ KDR^+^NKX2.5^low^ CPCs (Wang et al. 2021a, b) or MesP1^+^ CPCs (Neri et al. 2019), which are more likely to develop into heart valve endothelial cells. In the second stage, two or more signals, such as BMP, FGF, TGFβ, and NOTCH, are simultaneously activated to promote the generation of valvular endocardial cells (ECCs), which have been demonstrated to be the major origin of valvular cell (Lincoln et al. 2006). Subsequently, 80% of the cells become CD31^+^CD144^+^ VECs (Toshima et al. 2020). VEGF has been shown to induce endothelial cell fate (Gomez-Stallons et al. 2016). In the third stage, TGF-β, retinoic acid, BMP, or FGF is employed to trigger the endothelial-to-mesenchymal transition (EMT) to obtain VICs. BMP and epidermal growth factor (EGF) signaling play crucial roles in VICs proliferation and maturation. Matrices such as collagen hydrogels have been reported to promote the EMT process (Nachlas et al. 2018).

In the realm of hiPSC differentiation into valve cells, the yield of VECs and VICs remains relatively low. Single-cell sequencing data from human valves have revealed a high degree of heterogeneity in both endothelial and interstitial cells within calcified valves (Xu et al. 2020). However, due to the absence of specific cell markers, this issue has yet to be resolved. To address these challenges and advance the field, researchers are exploring various strategies. The development of novel single-cell sequencing techniques, spatial transcriptomics, and high-resolution mass spectrometry will provide a deeper understanding of valve development and calcification processes. Additionally, the integration of high-throughput compound screening and machine learning approaches could facilitate the identification of novel signaling pathways and small molecules that enhance hiPSCs differentiation into functional valve cells.

In conclusion, while challenges remain in the field of hiPSCs differentiation into valve cells, the advent of innovative technologies and the adoption of data-driven approaches hold promise for significant advancements. Overcoming these challenges will pave the way for the development of robust and efficient differentiation protocols, enabling the production of high-quality valve cells for regenerative medicine applications.

#### Calcific induce approaches

As key inducers of valve calcification, valve interstitial cells (VICs) undergo force stress and chemical stimuli that facilitate myofibroblast differentiation and osteogenic differentiation. Myofibroblast-like cells play a pivotal role in extracellular matrix remodeling during the pathogenesis of aortic valve calcification (Jian et al. 2003). TGFβ-1 is the most extensively studied factor in inducing α-SMA^+^ myofibroblast-like cells (Jian et al. 2003; Walker et al. 2004). This process is influenced by matrix stiffness (Gwanmesia et al. 2010; Rodriguez & Masters 2009). Regarding pro-osteogenic progression, there are two major mediums: 1) osteogenic medium: This medium includes dexamethasone, β-glycerophosphate, and L-ascorbic acid (AC) (Osman et al. 2006). β-Glycerophosphate serves as a phosphate source for bone mineral and induces osteogenic gene expression via extracellular related kinase phosphorylation; ascorbic acid facilitates osteogenic differentiation by increasing collagen type 1 secretion, and dexamethasone induces Runx2 expression, and Runx2-bone morphogenetic protein interaction is essential for osteogenic differentiation (Hamidouche et al. 2008; Kundu et al. 2009). 2) Pro-calcifying medium containing NaH_2_PO_4_ and ascorbic acid promotes inflammation and mineralization in VICs (Bouchareb et al. 2015; Goto et al. 2019). Other factors like LPS (Babu et al. 2008), VIC and VEC co-culture(Hjortnaes et al. 2015; Stadelmann et al. 2022) also activates inflammation and osteogenesis.

### 3D in vitro models of CAVD

Though VICs in culture appear to be the most relevant in vitro model for valve calcification, VICs are proved to undergo spontaneous activation in 2D culture (Benton et al. 2009a, b). Therefore, 3D in vitro CAVD models are actively explored. We classified 3D CAVD models into ex vivo model and 3D cell culture model by ECM composition, as summarized in Table 2.

**Table 2:** 3D in vitro models of CAVD

Classification	Type	Molding methods	Results	Advantages	Disadvantages
Ex vivo	valve leaflets (Weber et al. 2021)	AV leaflets from healthy 6–9 months Ovis; stretched with needles on silicon rubber rings; under pro-degenerative conditions for 14d-56d	At 14 d begins to form and at 56 d massive calcium accumulation in three layers by histologic staining; Col1A1, Col3A1, VIM, ACTA-2, OPN↑	easily applicable, reproducible, and cost effective; native valvular ECM and realistic VIC–VEC interactions	processed cultured under passive tension; regardless of cell types and factors in the blood circulation
	perfusion heart ( Kruithof et al. 2021)	whole mouse hearts (2–6 months) in MTCS; perfusion with osteogenic medium (OSM) or inorganic phosphates + Dex for 1 weeks	PI + Dex but not OSM induces valve leaflets calcification indicated by alizarin red-stainning; ALP and RUNX1/2/3 immunostaining; Endochondral differentiation staining	culture of mouse valves in their natural position in the heart and under specific hemodynamic conditions; exposing the leaflets to pro-calcific enviroment (relative native); shorten culture time	retrograde flow created a mechanical environment favoring calcification; regardless of cell types and factors in the blood circulation
Hydrogel-based 3D culture	Scaffold-based coculture (Hof et al. 2016)	Sheep aortic valves were decellularized and treated with trypsin or laser perforation, then reseeded with sheep VICs	low activation of repopulating VIC after 7 days of culture;MMP2,MMP9,αSMA↑	using fsL-mediated photodisruption to increase dECM permeability; short enzymatic treatment facilitate the migration of seeded VIC into the ECM	Not for CAVD modeling; Relatively limited interstital repopulation; hard to identify exact photodisrupted regions
	Scaffold-based coculture (Stadelmann et al. 2022)	A bilayer cryo-electrostatically spun scaffold, 6-y porcine VEC and VIC seeded onto FN-functionalized scaffold, cultured for 4 weeks in CM or OM	cell adherence, homogenous migration and proliferation↑; VICs interact with VECs↑; Runx2,SPP1↑;	a promising platform material to study calcification on a soft substrate;	could be integrated into perfusion or dynamic culture systems for studying disease progression
	3D stacked paper-based culture (Sapp et al. 2015)	6-m pVICs, filter paper layer printed with a wax-well plate template, implanted with a mixture of VICs and collagen	VIC migration↑; αSMA↑	Allows customization of the ECM and incorporates the ability to stack individual layers to control the thickness of the total culture	the position of cells in each layer cannot be controlled
	Hydrogel-based culture plateform (Porras et al. 2018)	pVICs seeded on either GelMA only or GelMA/GAG hydrogels (HA, CS), treated with 25 μg/mL human LDL or oxLDL for 72 h	GAGs enriched ECM leads to inflammatory↓, angiogenesis↑, deposition of oxidized lipoproteins↑	mimics enriched GAGs, quiescent VICs, and presence of lipoproteins in early CAVD	It does not include studies of the factors that regulate the onset of GAG enrichment or the importance of early features in fibrosis and calcification
	Micropatterning hydrogel based plateform (Duan et al. 2019)	12-year(Normal) or 75-y(CAVD) individual origin HAVIC were seeded on 3D micropatterned bioactive hydrogels consisting of Me-HA/Me-Gel, with a customized mask-guided photocrosslinking method;in OGM	αSMA↑, MMP-1↑, ALP↑; osteogenic differentiation↑in diseased HAVIC with patterning,	bioactive Me-HA/Me-Gel hydrogels with VIC ECM-like components and similar stiffness to the ventricularis and fibrosa layers of aortic heart valve leaflets	The width and space of the micropattern, and different degrees of alignment, were not studied
	Bioreactor model (Gould et al. 2012)	compression springs with gel inoculation, solidified for 60 min and seeded with porcine valve mesenchymal stromal cells	proliferation and apoptosis↑; F-actin↑; ACTA2↑	Implemented a novel bioreactor to investigate the relationship between anisotropic strain, cell differentiation, and matrix remodeling in 3D culture	It is unclear how cells interpret time- and direction-varying anisotropic strain fields in defined three-dimensional matrix structures
	Bioreactor culture (Ferdous et al. 2011)	HASMC and HAVIC obtained from non-sclerotic patients, molded in tubular collagen-cell mixture and cultured in pneumatic bioreactor with osteogenic media for 9 or 21 d	collagen I,MMP-2↑; calcium deposition↑; Runx2,ALP,αSMA↑; HASMCs expresses higher osteogenic markers and matrix remodeling than HAVICs	Comparing vascular versus valvular calcification with tissue-engineered collagen gels	Could not interprete regional differences due to mechanical force variation
	VICs 3D culture (Lim et al. 2016)	mVICs were encapsulated in 2 mg/mL collagen, treated with 5 mmol/L β-GP, and 50 μg/mL of ascorbic acid in α-MEM	thickness, calcification↑; fibronectin, a-SMA, collagen receptor, discoidin domain tyrosine kinase receptor 2↑; F-actin, NF-κB, JNK↑	linking inflammation with the clinical features of aortic stenosis: valvular retraction, stiffening, and formation of calcified nodules	Inflammatory factors are limited to TNF-α
	VICs 3D culture (Hjortnaes et al., 2015)	10-m pVICs cultured in 1% HAMA-5% GelMA, treated by TGFβ	vimentin, αSMA, MMP9, Col1A1↑	enlable to maintain a quiescent VIC phenotype before stimulation	Hydrogel platforms are not in an environment of repetitive strain and pressure
	VECs and VICs co-culture (Gee et al. 2021)	pVECs and pVICs seeded in a mechanically constrained collagen alone or in co-culture configurations	cell and matrix aggregates↑; αSMA,pSAMD2,ACTA2↑; SOX9↓	The model supports its use to test mechanisms of intercellular communication in valves and their pharmacological control	mechanical characterization of collagen gel is not feasible
	VECs and VICs co-culture (Bramsen et al. 2022)	6–8 m pAVICs were seeded into and pAVEC were seeded on top of hydrogels with different collagen composition (Con:1.5 mg/mL; stiff: 2.2 mg/mL; with CS: 1.5 mg/mL collagen + 20 mg/mL CS; + HA:1.5 mg/mL collagen + 20 mg/mL HA) for 2w	αSMA,ALP↑; cellular invasion rates↑; proliferation activity↑	GAGs (CS and HA) mimic altered ECM (matrix mineralization situation) to study EndMT-derived aVIC activity	Gender was not addressed
	VECs and VICs co-culture (Vadana et al. 2020)	VICs and VECs from calcified human, Gelatin-based 3D constructs with VIC encapsulated in hydrogel and VEC seeded on top, exposed to osteogenic medium (10 mmol/L β-GP, 10 ng/mL ascorbic acid and 10^−8^ mol/L dexamethasone)	αSMA↓; vimentin-; MMP1,MMP13,MMP2, MMP9↑; Runx2, OCN,OPN↑; HG trigger BMP and TGF-β signaling further	3D model with human valvular cells plus high glucose imply the increased risk of degenerative aortic valve disease and calcification found in diabetic patients	Lack of flow-induced shear stress and hemodynamic forces
	3D-bioprinting (Immohr et al. 2022)	oVICs dissolved in a hydrogel composed of 2% alginate and 8% gelatin, 3D bioprinting and incubated for 28d	cell viability↑	reduce associated VIC damage and increase long-term cell viability	cannot establish 3D-bioprinting of even more vulnerable aortic valvular endothelial cells
	3D-bioprinting (Immohr et al. 2023)	oVICs and VECs, dissolved in a hydrogel consisting of 2% alginate and 8% gelatin, 3D bioprinting with architectures	cell viability↑	first 3D-bioprinted AV model combining both VIC and VEC in a single multicellular construct	unintentional concomitant induction of endothelial-to-mesenchymal transition

#### Ex vivo CAVD model

Ex vivo CAVD models aim to preserve the intact valve structure in vitro, providing direct evidence to elucidate the structural destruction and calcification induced by mechanical and metabolic factors. Valves can be subjected to various biochemical and hemodynamic stimuli to explore their role in cardiac valve remodeling. Utilizing the Miniature Tissue Culture System (MTCS) (Kruithof et al. 2015), researchers perfused fresh mouse hearts in vitro, demonstrating that inorganic phosphate plus Dex, not osteogenic medium, induced valve leaflet calcification (Kruithof et al. 2021). Pig aortic valves sewn into the heart valve chamber of a bioreactor system and cultured in pro-degenerative medium for 7 days exhibited significant leaflet calcification (Niazy et al. 2021). In contrast, mechanical stress is more commonly employed in ex vivo models. Rat aortic valves cultured in a bioreactor flow culture system to replicate normal valve cycling, with constant opening and closing, altered gene expression profiles associated with valve remodeling or repair (Maeda et al. 2016) designed a device that simultaneously exposes both surfaces of aortic valve leaflets to their native side-specific shear stress, elucidating the role of fluid shear stress, maintaining leaflet structure, cell viability, and cell proliferation for the intended culture duration of 96 h (Sun et al. 2011).

Ex vivo CAVD models often require complex equipment and incur significant costs, making them susceptible to contamination or loss of sterility. Moreover, the intrinsic complexity of tensional bioreactor systems leads to highly variable outcomes, compromising the reliability of study results. As the convergence of biomedical and tissue engineering technologies progresses, the development of ex vivo calcification models will evolve towards greater precision and standardization, while also increasing in complexity to accurately replicate the pathophysiological environment of human valves, including the presence of inflammatory cell infiltrates. These advancements will provide essential support for elucidating the mechanisms of valve calcification and paving the way for novel therapeutic strategies.

#### 3D cell culture model

##### Scaffold-based 3D system

Apart from ex vivo models that preserve the valve's native ECM, constructing 3D cell culture models of VICs and/or VECs using various matrices appear to be a more straightforward and practical approach. Based on the matrix type, we can categorize 3D culture models into scaffold-based co-culture systems and hydrogel-based systems. Scaffolds can be derived from decellularized ECM or non-hydrogel synthetic materials. Decellularized sheep aortic valves treated with trypsin or fsL-mediated photodisruption to enhance dECM permeability, when reseeded with sheep VICs, can potentially recreate the native valve's cell-ECM interactions (Sun et al. 2011). However, improving interstitial repopulation in detergent-derived dECM remains challenging. Another study used cryo-electrostatic spinning technology to construct a bilayer scaffold that was encoded with fibronectin to support the growth of porcine VICs and VECs. The scaffold was then treated with osteogenic medium, which resulted in increased cell interaction and the expression of Runx2 and SPP1 (Stadelmann et al. 2022).

##### Hydrogel-based 3D culture model

Three-dimensional (3D) cell culture systems, particularly those employing naturally derived extracellular matrix (ECM) polymers, offer a promising approach to mimicking the microenvironment of native valve tissue and studying the behavior of valve cells (Hjortnaes et al. 2016). Hydrogels are broadly categorized into natural hydrogels, synthetic hydrogels, and hybrid hydrogels. Natural hydrogels exhibit excellent biocompatibility but are limited in mechanical properties like stiffness and stretchability. For instance, collagen hydrogels are widely employed for the 3D culture of valve interstitial cells (VICs) due to their ability to support cell spreading and proliferation (Gee et al. 2021; Hof et al. 2016; Lim et al. 2016; Sapp et al. 2015). However, collagen hydrogels are prone to shrinkage and may not provide adequate mechanical support (Hof et al. 2016). Synthetic hydrogels achieve superior mechanical properties through chemical cross-linking, but their biocompatibility may be compromised. Hybrid hydrogels, which combine natural and synthetic biomaterials, have emerged as promising ECM analogues for studying VICs behavior in 3D microenvironments. VICs cultured in 3D methacrylic hyaluronic acid (Me-HA) hydrogels exhibit restricted spreading morphology in the absence of cell adhesion motifs (Duan et al. 2013). Additionally, methacrylic gelatin (Me-Gel) hydrogel allows VICs to retain their native morphology (Benton et al. 2009a, b). Hybrid hydrogels employed in CAVD tissue construction include collagen-Glycosaminoglycans(GAGs) (Bramsen et al. 2022), HAMA-GelMA (Hjortnaes et al. 2016; Meerman et al. 2021), Gelatin methacrylation/GAG (Porras et al. 2018), Me-HA-Me-Gel (Duan et al. 2019), and matrigel-collagen (Lam et al. 2019) hydrogel. These hybrid hydrogels offer a balance of biocompatibility and mechanical properties that better recapitulate the native valve tissue microenvironment. Additionally, hydrogels can be fabricated into shaped substrates to induce valve calcification. Duan et al. employed a custom photomask-guided photo-cross-linking technique with human aortic valve interstitial cells (HAVICs) to generate 3D micropatterned bioactive hydrogels, demonstrating that striped micropatterning promotes osteogenic differentiation of diseased HAVICs in osteogenic medium (Duan et al. 2019).

With regard to cell selection for CAVD 3D culture models, hydrogels are most commonly mixed with VICs, and then induction conditions, such as osteogenic medium, TNF-α, and TGFβ, are added to stimulate calcification formation and prevent spontaneous activation of VICs in planar culture (Lim et al. 2016; Hjortnaes et al. 2015). To further elucidate the interactions between VECs and VICs in CAVD, researchers have employed various in vitro culture models. One approach involves co-seeding VECs and VICs within mechanically constrained collagen hydrogels (Gee et al. 2021). Alternatively, VICs can be encapsulated within a hydrogel, with VECs seeded on top (Bramsen et al. 2022). By manipulating the hydrogel stiffness (Bramsen et al. 2022) or exposing the cells to osteogenic medium (Vadana et al. 2020), the formation of calcified nodules can be enhanced, facilitating the study of VECs-VICs interactions under these conditions. Grande-Allen's study, which cultured porcine aortic valve interstitial cells (PAVICs) and porcine aortic valve endothelial cells (PAVECs) within collagen I hydrogels containing the GAGs, chondroitin sulfate (CS) or hyaluronic acid (HA), demonstrated that CS enhanced the formation of calcified nodules even in the absence of osteogenic culture medium (Grande-Allen et al. 2007). These methods have provided valuable insights into cell–cell interactions in the context of CAVD. 3D bioprinting technology has emerged as a valuable tool for generating 3D valve culture systems. Immohr et al. investigated optimal printing and culture parameters and hydrogel composition for 3D bioprinting of VICs (Immohr et al. 2022, 2023). They proved that using DMEM-based hydrogels can significantly improve the long-term cell viability and overcome printing-related cell damage.

In addition to cellular considerations, shape and function mimicry are also crucial aspects of in vitro valve and calcification modeling. Sapp et al. seeded VICs within a 3D stacked paper-based system to effectively replicate the full thickness of native valve tissue (Sapp et al. 2015). Other approaches utilize functional bioreactors with engineering technology to investigate the effects of mechanical strain on valvular interstitial cells (VICs) and the underlying molecular pathways involved in valve calcification. For instance, pig VICs mixed with type I collagen inoculated into an anisotropic biaxial strain bioreactor exhibited time-dependent VICs orientation and collagen fiber alignment (Gould et al. 2012). Another study combined collagen I, human aortic smooth mucle cells (HASMCs), and HAVICs with osteogenic conditions to evaluate the effect of cyclic strain on calcification (Ferdous et al. 2011). Mendoza et al. developed a three-dimensional microfluidic device of the aortic valve fibrosa to study the effects of shear stress and endothelial cell presence on calcification (Mendoza et al. 2022).Other type is using functional bioreactors with engineering technology, to investigate the effects of mechanical strain on valvular interstitial cells (VICs) and the underlying molecular pathways involved in valve calcification. Pig VICs mixed with type I collagen inoculated into anisotropic biaxial strain bioreactor causes time dependent VICs orientation and collagen fiber alignment (Gould et al. 2012). Another research combined collagen I, HASMCs and HAVICs with osteogenic conditions to evaluate effect of cyclic strain on calcification (Ferdous et al. 2011). Furthermore, Mendoza et al. developed a three-dimensional microfluidic device of the aortic valve fibrosa to study the effects of shear stress and endothelial cell presence on calcification (Mendoza et al. 2022).

Despite relatively rapid development of in vitro 3D models of CAVD in recent years, there are still some limitations. Firstly, current hydrogel systems fail to replicate the composition and high cell activity of the native extracellular matrix, necessitating further experimentation and refinement. Secondly, due to the inherent nature of gels, diffusion must be considered when applying risk factors stimulation. Thirdly, simulating mechanical stimulation of valve tissue remains a challenge. Overall, 3D aortic valve calcification tissue models are still in their nascent stages, but their potential applications hold immense promise. With the continuous advancement of interdisciplinary integration, innovation in material science, and the refinement of valve cell differentiation and 3D printing technologies, 3D tissue models are poised to become indispensable tools for CAVD research and treatment.

## Conclusions

CAVD is the most common valvular heart disease, primarily affecting the elderly population. As the aging problem worsens, the incidence of CAVD is expected to rise significantly. Establishing reliable CAVD models is crucial for advancing our understanding of the disease's pathogenesis and identifying effective treatment strategies. The development of CAVD models has undergone significant advancements over the past two decades. Animal models have played a pivotal role in elucidating the disease's intricacies and have reached a considerable level of maturity. Transgenic mice models, combined with high-fat diet feeding or mechanical injury, allow for in-depth analysis of the impact of target genes on CAVD progression. Larger animal models, such as pigs, offer closer resemblance to human valve structure and pathophysiological responses. Each species, including mice, rabbits, and pigs, possesses unique advantages, and these animal models have made substantial contributions to our understanding of CAVD pathogenesis. However, they face limitations in clearly defining the distinct stages of CAVD disease and overcoming phenotypic differences arising from species variations.

In vitro models, utilizing valve interstitial cells (VICs), have provided valuable insights into CAVD pathogenesis by examining the effects of various stimuli, such as chemical factors, shear stress, and cyclic strain. The advantage of in vitro models lies in their ability to rapidly and precisely manipulate a large number of variables. Additionally, iPSCs-based differentiation technology holds promise for generating large quantities of human valve cells. However, VICs in flat culture have a tendency to spontaneously activate, and simulating the calcification changes induced by stress shock in the valve is challenging at the monolayer cell level. In response to these limitations, 3D culture systems, including bioreactors, microfluidic systems, and hydrogel hybrid systems, have emerged as promising advancements in CAVD in vitro disease modeling. These systems offer a more realistic representation of the complex microenvironment of the native aortic valve, enabling a more comprehensive assessment of cellular interactions and responses.

Despite the shortcomings of various models, the intersection of disciplines and the emergence of new technologies hold immense potential to further refine CAVD models. By addressing these limitations and embracing innovative approaches, researchers can gradually unravel the intricacies of CAVD and ultimately pave the way for the development of effective therapies to prevent and mitigate the disease.

## Data Availability

Not applicable.

## Abbreviations

3D: Three-Dimensional
AC: Ascorbic Acid
AV: Aortic Valve
AVICs: Aortic Valvular Interstitial Cells
AVS: Aortic Valve Stenosis
AVWI: Aortic Valve Wire Injury
CAVD: Calcific Aortic Valve Disease
CPCs: Cardiac Progenitor Cells
CS: Chondroitin Sulfate
dECM: Decellularized Extracellular Matrix
ECM: Extracellular Matrix
EGF: Epidermal Growth Factor
EndoMT: Endothelial-to-Mesenchymal Transition
GAGs: Glycosaminoglycans
HA: Hyaluronic Acid
HASMCs: Human Aortic Smooth Muscle Cells
HAVICs: Human Aortic Valve Interstitial Cells
HFD: High-Fat Diet
HFCD: High-Fat and Choline Diet
hiPSC: Human induced Pluripotent Stem Cells
LPS: Lipopolysaccharide
Me-Gel: Methacrylic Gelatin
Me-HA: Methacrylic Hyaluronic Acid
MTCS: Miniature Tissue Culture System
PAVECs: Porcine Aortic Valve Endothelial Cells
PAVICs: Porcine Aortic Valve Interstitial Cells
PSCs: Pluripotent Stem Cells
SMCs: Smooth Muscle Cells
VECs: Valve Endothelial Cells
VICs: Valve Interstitial Cells
WHHL: Watanabe Heritable Hyperlipidemic
